# Altered glycosylation profiles of serum IgG in Takayasu arteritis

**DOI:** 10.1186/s40001-023-01035-4

**Published:** 2023-02-08

**Authors:** Lingyu Liu, Jing Li, Yunjiao Yang, Chaojun Hu, Xinping Tian

**Affiliations:** Department of Rheumatology and Clinical Immunology, Chinese Academy of Medical Sciences & Peking Union Medical College, National Clinical Research Center for Dermatologic and Immunologic Diseases (NCRC-DID), Ministry of Science & Technology, State Key Laboratory of Complex Severe and Rare Diseases, Peking Union Medical College Hospital (PUMCH), Key Laboratory of Rheumatology and Clinical Immunology, Ministry of Education, Beijing, China

**Keywords:** Takayasu arteritis, Immunoglobulin G, Glycosylation, Lectin microarray

## Abstract

**Background:**

Takayasu arteritis (TAK) is an autoimmune inflammatory disorder with an undefined etiology. This study aimed to characterize the glycosylation profiles of serum immunoglobulin G (IgG) in patients with TAK.

**Methods:**

Lectin microarrays containing 56 types of lectins were used to detect the glycan levels of serum IgG in 164 patients with TAK, 128 patients with atherosclerosis used as disease controls (DCs), and 100 healthy controls (HCs). Differentially altered glycosylation patterns between TAK and control groups as well as between TAK subgroups were identified and further validated by lectin blot. The classification performance of the TAK-specific glycosylation change was measured by receiver-operating characteristic (ROC) curve analysis.

**Results:**

Lectin microarray analysis revealed significantly increased *N*-Acetylgalactosamine (GalNAc) levels in the TAK group compared to the DC and HC groups (all *p* < 0.01). For TAK subgroups, significantly decreased mannosylation was observed in patients with active TAK compared to patients with inactive disease (*p* < 0.01). These differences were validated by lectin blot. In addition, GalNAc levels exhibited a considerable potential for discriminating patients with TAK from patients with atherosclerosis, with an area under the curve of 0.749 (*p* < 0.001), a sensitivity of 71.7%, and a specificity of 73.8%.

**Conclusions:**

Serum IgG in patients with TAK displayed disease-specific glycosylation alterations. Aberrant GalNAc glycosylation showed substantial value as a diagnostic biomarker. The potential proinflammatory properties of the abnormal glycans may provide new insights into the role of humoral immunity in the pathogenesis of TAK.

**Supplementary Information:**

The online version contains supplementary material available at 10.1186/s40001-023-01035-4.

## Background

Takayasu arteritis (TAK) is an immune-mediated chronic inflammatory disease that mainly involves the aorta and its major branches. It predominantly affects young women and is more prevalent in Asian and North African countries, but cases can also be seen in the rest of the world [1]. Vessel wall thickening, lumen stenosis and occlusion are common features of TAK. The carotid and vertebral arteries, and arteries of the upper extremities are frequently affected, leading to ischemic complications, such as vertigo, stroke, and intermittent upper limb weakness [2]. These ischemic manifestations due to arterial stenosis caused by vasculitis are quite similar in clinical signs, symptoms and radiological findings to those caused by atherosclerosis [3]. Therefore, atherosclerosis should be excluded in the differential diagnosis when evaluating older patients with suspected large vessel vasculitis. However, biological indicators for discriminating arterial stenosis caused by vasculitis from that caused by atherosclerosis are still lacking.

In addition, the mechanism underlying the development of TAK remains elusive. The critical role of cellular immunity in TAK, represented by CD4 + T cells, has been explored in great detail in previous studies [4], but the effects of antibody-mediated humoral immunity have not yet been fully unraveled. Deposition of immunoglobulin G (IgG) in the intima of involved arteries has been observed in patients with TAK [5]. Moreover, the presence of anti-endothelial cell antibodies (AECAs) in patients with TAK has shed new light on the role of humoral immunity in the pathophysiological mechanisms of TAK [6]. Nevertheless, further studies are needed to clarify how B cells and autoantibodies contribute to the disease pathogenesis.

Glycosylation is one of the most important post-translational modifications during the process of protein biosynthesis, which influences biological functions vastly by altering the structure and stability of glycoproteins [7]. IgG is known to be the most abundant glycoprotein in the human serum. Multiple studies have demonstrated that the glycosylation of IgG varies significantly under different physiological and pathological circumstances, especially in some inflammatory autoimmune diseases [8]. For instance, altered IgG glycosylation has been observed in rheumatoid arthritis (RA) [9], systemic lupus erythematosus (SLE) [10], Sjogren’s syndrome [11], multiple sclerosis [12], inflammatory bowel disease [13], and Lambert-Eaton myasthenic syndrome [14]. At present, no study has reported the glycosylation changes of serum IgG in TAK.

Lectins are proteins that can bind specifically to certain monosaccharides or oligosaccharides. The recently developed lectin microarray is a novel tool for glycan analysis that enables obtaining global glycosylation patterns in a rapid and highly sensitive way without the need for releasing glycans [15]. In this study, we utilized lectin microarrays to depict the glycosylation patterns of serum IgG in patients with TAK and search for a potential diagnostic marker that can be used to distinguish TAK from atherosclerosis. Meanwhile, we investigated differences in IgG glycosylation in different disease states of TAK, aiming to provide new insights into the role of IgG glycosylation in the pathogenesis of TAK.

## Methods

### Patients and samples

All serum samples in this study were collected at Peking Union Medical College Hospital (PUMCH). In total, 392 serum samples collected from 164 patients with TAK, 128 patients with peripheral arterial disease (PAD), and 100 healthy controls (HCs) were used for lectin microarray analysis. The inclusion criteria required every participant in this study to be older than 18 years on the sampling points. Patients with other autoimmune diseases, active infections, pregnancies, cancers, or any severe comorbidities were excluded. For TAK group, patients who met the 1990 American College of Rheumatology classification criteria for TAK were included [16], and patients with incomplete laboratory data on the sampling points were excluded. Patients with TAK were further divided into inactive (*n* = 82) and active (*n* = 82) groups with matched age and gender, according to the 1994 National Institutes of Health criteria [17]. Since PAD is also a chronic disease characterized by stenosis or occlusion of arteries but with an atherosclerotic etiology other than vasculitis [18], PAD patients were taken as disease controls (DCs), which includes 75 patients with carotid artery stenosis (CAS) and 53 with chronic limb-threatening ischemia (CLTI). Patients in DC group should meet the 2017 European Society of Cardiology diagnostic criteria for PAD [19], and patients with arterial stenosis/occlusion secondary to non-atherosclerotic conditions (i.e., Buerger's disease) were excluded. For HCs, healthy subjects from the health examination center matched for age and gender to DC group were included.

Specific glycosylation changes were validated by lectin blot in a smaller cohort consisting of 48 patients with TAK (24 inactive and 24 active), 16 DCs (8 CAS and 8 CLTI), and 16 HCs, of which some patients were from the previous study cohort (10 inactive TAK, 10 active TAK, 6 DCs), and the others came from a new set of patients. To reduce the bias caused by age differences in the previous cohort, 16 new HCs were included in the validation cohort to match to the TAK group. The gender distribution among the three groups in the validation cohort was also made consistent. Serum was separated from the whole blood specimen and stored at − 80 °C until use. This study was approved by the Institutional Review Board of PUMCH, Beijing, China (JS-2038). Written informed consent was obtained from each participant and the study was conducted following the Declaration of Helsinki.

### Lectin microarray

To detect the glycosylation patterns of serum IgG, a commercial lectin microarray chip (BCBIO Biotech, Guangzhou, China) with triplicate spots of 56 types of lectins was used (see Additional file 1: Fig. S1 for the layout of the lectin microarray). The 56 types of lectins were known to bind specifically to common glycan structures in mammals (see Additional file 3: Table S1 for the list of lectins). Lectin microarray chips at -80 °C storage were first equilibrated at 4 °C and warmed at room temperature for 30 min before use, and then the chips were incubated in blocking buffer (1% BSA in PBS) for 2 h. After washing with PBS, the microarray chips were dried by centrifugation. The serum from each sample was diluted 1:200 in 0.05% Tween 20 (PBST) and 200 µl of each diluted sample were applied to one well on the chips, and then the chips were incubated at 4 °C overnight. Afterward, the chips were washed with PBST five times and incubated with 3 ml Alexa Fluor 647-labeled goat anti-human IgG antibody (1:1,000; Jackson ImmunoResearch) for 45 min at room temperature protected from light. After washing thoroughly with PBST and ddH2O, the chips were dried by centrifugation and scanned with a GenePix 4000B Microarray Scanner (Molecular Devices, Sunnyvale, CA) at a wavelength of 647 nm. The photomultiplier tube gain was set to 600.

### Lectin microarray data analysis

The fluorescent images were converted to a digital format for subsequent analysis by extracting the median foreground and background intensity values of each spot on the chips using the GenePix Pro 6.0 software (Molecular Devices, Sunnyvale, CA). The signal-to-noise ratio (S/N) of each lectin spot, defined as the ratio of the median signal intensity of the foreground to the background, was calculated. To minimize the inter-array bias, we used the method of Silver et al. [20] to normalize the S/N data. Significant differences in lectin-binding levels among groups were determined according to the methods described by Hu et al. [21]. A significant difference was considered to be present when the following two conditions were satisfied: (a) the fold change (FC) between two groups [group1 (S/N)/group2 (S/N)] > 1.3 or < 0.77; (b) *P*-value < 0.05.

### Lectin blot and Western blot

Since the glycans of IgG known to alter the conformation and function are attached to the two heavy chains [22], the IgG heavy chains (50 kDa) are chosen to verify the microarray results in lectin blot [23]. First, serum proteins at 1:100 dilution were separated by 10% sodium dodecyl sulfate–polyacrylamide gel electrophoresis (SDS–PAGE) and transferred onto polyvinylidene fluoride membranes (Millipore, Billerica, MA). After being blocked with blocking buffer (QuickBlock™ Blocking Buffer for Western Blot, Beyotime Biotechnology, Nanjing, China) at room temperature for 30 min, the membranes were incubated with 20 μg/ml of Cy3 (GE Healthcare)-labeled glycine max lectin (SBA; Vector Laboratories Inc., US), Concanavalin A lectin (ConA; Vector Laboratories Inc., US), and Morniga M lectin (MNA-M; EY Laboratories, Inc., US) overnight at 4 °C protected from light. Excess lectins were washed away with TBST. Finally, the membranes were visualized by a fluorescence signal system of Typhoon FLA 9500 (GE Healthcare). Serum proteins from the same healthy subject were used as a reference. In addition, to confirm the location of IgG in immunoblotting and exclude the influence of different IgG concentrations on the measurements of IgG glycosylation, Western blotting of serum IgG was performed. After blocking unspecific binding sites, the membranes were incubated with goat anti-human IgG (H + L) -HRP conjugated antibody (1:15,000; EASYBIO, China) at room temperature for 1 h. Protein bands were visualized by ImageQuant software (GE Healthcare). The signal intensity was quantified for further analysis by ImageJ software (National Institutes of Health, Bethesda, MD, USA). The intensity ratios (lectin/IgG) were calculated.

### Statistical analysis

Continuous variables were presented as mean ± standard deviation or median [interquartile range (IQR)], where appropriate. Categorical variables were presented as numbers (percentages). Multiple group comparisons were performed using one-way ANOVA or Kruskal–Wallis test where appropriate. Mann–Whitney U test or Student’s t-test was applied to compare between two groups as appropriate. Spearman’s rank correlation coefficient was used to evaluate the relationship between the glycan levels of IgG and laboratory results. A *P*-value < 0.05 was considered statistically significant. To evaluate the diagnostic performance of differentially altered glycan levels, a receiver-operating characteristic (ROC) curve analysis was carried out and the area under the ROC curve (AUC) was calculated. The optimal cutoff value was determined by the ROC curve and the corresponding sensitivity and specificity were measured. SPSS 22.0 (SPSS 22.0 for Windows, SPSS, Chicago, Illinois, USA) and GraphPad Prism 8 (GraphPad Software, La Jolla, CA) were used to conduct the statistical analyses, and GraphPad Prism 8 and R software (version 4.0.5) were used for plotting graphics.

## Results

### Demographic and clinical characteristics of subjects

The basic characteristics of 392 participants included in the lectin microarray analysis are summarized in Table 1. As shown in the table, no significant difference was found in age or gender distribution between the inactive and active TAK groups. However, there were differences in age and gender distribution between the TAK group and the other two groups. The relatively late onset of PAD probably explains the disparity in age distribution. In addition, the levels of all measured inflammatory indicators including hypersensitive C-reactive protein (hsCRP), erythrocyte sedimentation rate (ESR), interleukin-6 (IL-6), and tumor necrosis factor-alpha (TNF-α) were significantly higher in the active TAK group than in the inactive TAK group (*p* < 0.01). No significant difference was observed in disease duration, ongoing glucocorticoids, and ongoing disease-modifying antirheumatic drugs (DMARDs) and/or biological DMARDs (bDMARDs) between the inactive and active TAK groups. In the lectin blot analysis, the mean age was 31.75 ± 8.43 years for patients with TAK and 33.38 ± 5.66 years for HCs.

**Table 1:** Clinical characteristics of subjects in the lectin microarray cohort

	TAK (*n* = 164)		DCs (*n* = 128)		HCs (*n* = 100)
	Inactive (*n* = 82)	Active (*n* = 82)	CAS (*n* = 75)	CLTI (*n* = 53)	
Male/Female	9/73	9/73	63/12	44/9	81/19
Age (year)^a^	31 (27, 37)	32 (27, 35)	66 (61, 71)	68 (62, 74)	65 (63, 68)
Laboratory results^a^					
hsCRP (mg/L)^b^	0.9 (0.3, 1.9)	10.4 (2.0, 36.5)	/	/	/
ESR (mm/h)^b^	8.0 (3.8, 12.3)	20.0 (8.0, 49.3)	/	/	/
IL-6 (pg/ml)^b^	2.8 (2.0, 4.5)	8.2 (2.7, 16.7)	/	/	/
TNF-α (pg/ml)^b^	5.5 (4.6, 6.7)	7.2 (5.4, 18.2)	/	/	/
Disease duration (month)^a^	78.0 (56.6, 119.0)	68.1 (27.5, 117.7)	/	/	/
Ongoing glucocorticoids, *n* (%)	61 (74.4)	63 (76.8)	/	/	/
Ongoing (b)DMARDs, *n* (%)	72 (87.8)	68 (82.9)	/	/	/

*TAK* Takayasu arteritis, *DCs* disease controls, *HCs* healthy controls; *CAS* carotid artery stenosis, *CLTI* chronic limb-threatening ischemia, *hsCRP* hypersensitive C-reactive protein, *ESR* erythrocyte sedimentation rate; IL-6, interleukin-6, *TNF-α* tumor necrosis factor-alpha; (b)DMARDs, (biological) disease-modifying antirheumatic drugs

^a^Median [interquartile range (IQR)]; ^b^*p* < 0.01, inactive TAK group vs. active TAK group

### Serum IgG glycosylation patterns detected by lectin microarrays

**Fig. 1:**
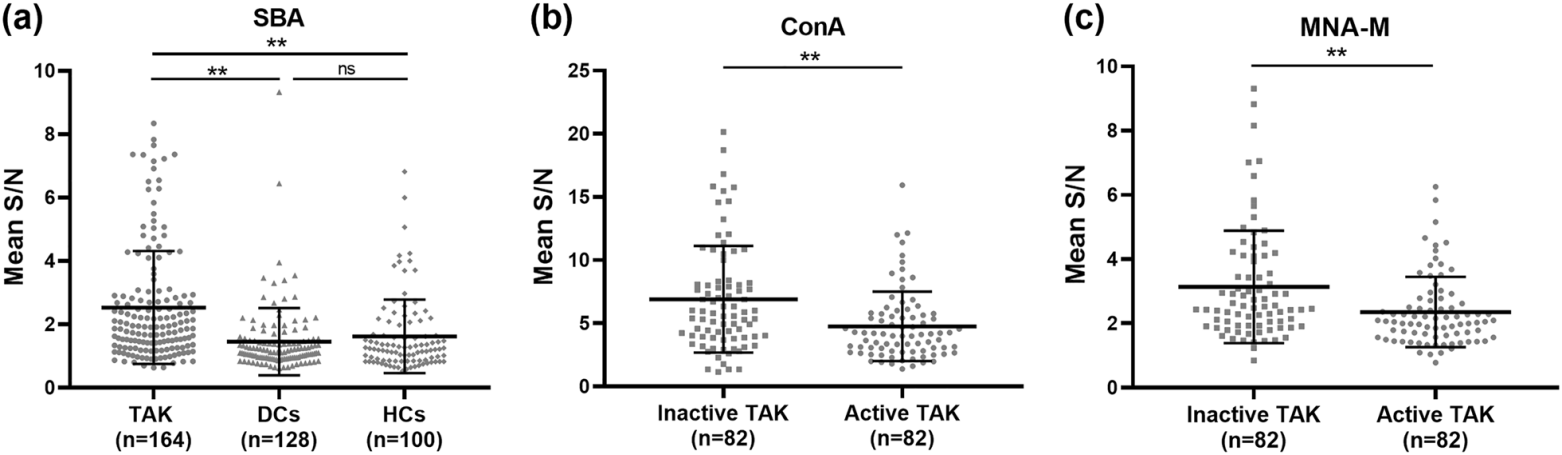
Differentially altered glycosylation patterns of serum IgG detected by lectin microarray. **a** comparisons of serum IgG glycopatterns among patients with Takayasu arteritis (TAK), disease controls (DCs) and healthy controls (HCs); **b** and **c** comparisons of serum IgG glycopatterns between patients with inactive and active TAK. **p < 0.001; *ns* not significant, *SBA* Glycine max lectin; *ConA* Concanavalin A lectin; *MNA-M* Morniga M lectin, *S/N* signal-to-noise ratio

To investigate the glycosylation profiles of serum IgG in TAK, lectin microarrays were used, which enabled a high-throughput and ultrasensitive detection of the glycoforms. Serum samples from 164 patients with TAK, 128 DCs, and 100 HCs were tested. After normalizing the signal values, only one lectin showed a significant difference among the three groups. As shown in Fig. 1, there is a significantly higher signal intensity of SBA in the TAK group compared to DCs and HCs (FC = 1.97, *p* < 0.0001; FC = 1.77, *p* < 0.0001, respectively), indicating increased glycan levels of *N*-Acetylgalactosamine (GalNAc) of serum IgG in patients with TAK. No significant difference was found between DCs and HCs (*p* > 0.99). In addition, decreased mannose levels, as implicated by less binding to ConA (FC = 0.69, *p* = 0.0002) and MNA-M (FC = 0.69, *p* = 0.0005), were found in patients with active TAK relative to patients with inactive disease.

### Validation of glycosylation changes of serum IgG by lectin blot

**Fig. 2:**
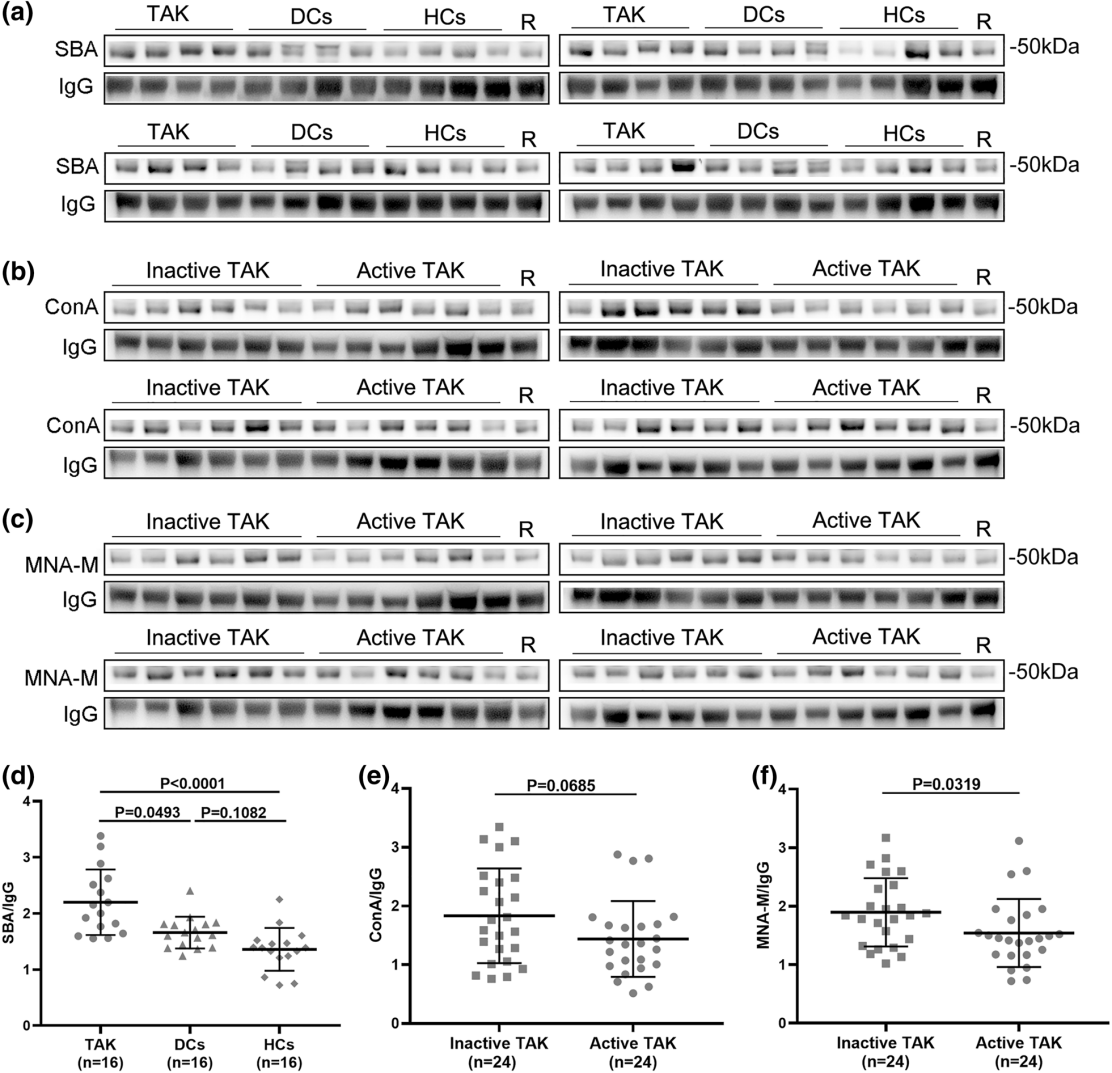
Validation of differentially binding lectins among different groups by lectin blot. **a** and **d** Lectin blot of SBA for serum IgG was performed in 16 patients with TAK (8 inactive and 8 active), 16 DCs, and 16 HCs. The signal intensity of IgG measured by Western blot was used as an internal control. The fluorescence intensity of the lectin blot band of the same healthy subject was used as a reference to correct for differences among membranes. The expression of SBA signal was normalized to IgG. The SBA/IgG of the TAK group was significantly higher compared with that of DCs and HCs. **b** and **e** Lectin blot of ConA for serum IgG between patients with inactive (*n* = 24) and active (*n* = 24) TAK. The ConA/IgG showed no significant difference between patients with inactive and active TAK (*p* = 0.0685). **c** and **f** Lectin blot of MNA-M for serum IgG between patients with inactive (*n* = 24) and active (*n* = 24) TAK. The MNA-M/IgG in the active TAK group was significantly lower than that in the inactive TAK group (*p* = 0.0319). SBA, Glycine max lectin; ConA, Concanavalin A lectin; MNA-M, Morniga M lectin; R reference

As shown in Fig. 2, the intensity ratios (SBA/IgG) in the TAK group were significantly higher than that in DCs and HCs (*p* = 0.0493 and *p* < 0.0001, respectively) and no significant difference was found between DCs and HCs (*p* = 0.1082), which were consistent with the results of the lectin microarray analysis. For TAK subgroups, MNA-M/IgG was significantly lower in the active TAK group than in the inactive TAK group (*p* = 0.0319). However, no significant difference was observed in ConA/IgG between the inactive and active TAK groups (*p* = 0.0685), although a similar decreasing trend was shown. Overall, these results confirmed the reliability of the lectin microarray test. Furthermore, correlation analysis showed a significant negative correlation between the signal intensities of MNA-M, as well as ConA, detected by lectin microarrays and hsCRP, ESR, and IL-6 (MNA-M: *r* = − 0.230, *p* = 0.003 for hsCRP, *r* = − 0.221, *p* = 0.005 for ESR, and *r* = − 0.184, *p* = 0.020 for IL-6, respectively; ConA: *r* = − 0.192, *p* = 0.014 for hsCRP, *r* = − 0.155, *p* = 0.047 for ESR, and *r* = − 0.242, *p* = 0.002 for IL-6, respectively). For TNF-α, the correlation was also negative (*r* = − 0.155 for MNA-M and *r* = − 0.149 for ConA, respectively), but not significant (*p* = 0.051 for MNA-M and p = 0.060 for ConA). These indicated a potential relationship between the mannose level of serum IgG and these inflammatory indicators. In addition, the MNA-M signal intensities between patients with and without ongoing glucocorticoids, and DMARD and/or bDMARDs in TAK group, were compared. No significant difference was found in the subgroup analysis (Additional file 2: Fig. S2).

### Evaluation of the capability of SBA to differentiate TAK from PAD

**Fig. 3:**
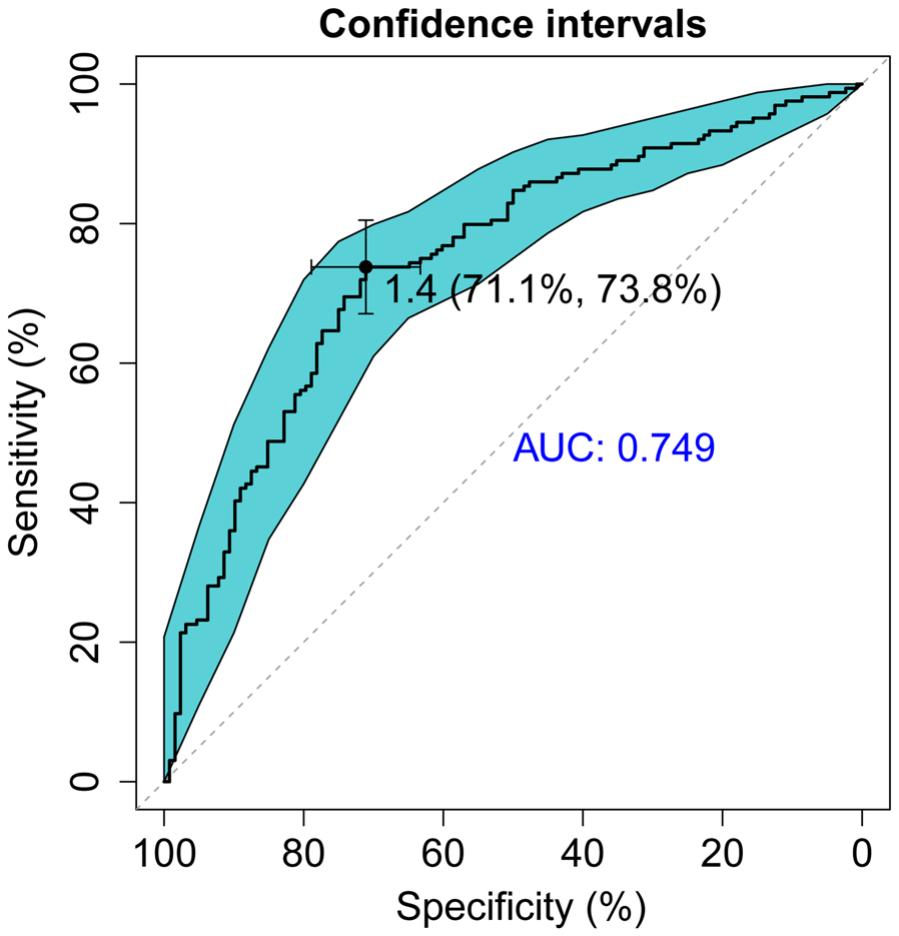
Receiver operating characteristic (ROC) curve analysis of SBA signal intensities from lectin microarrays. DeLong method was used to calculate the confidence intervals of ROC curve. The best predictive value of SBA signal intensity (1.4) and the corresponding specificity and sensitivity (71.1% and 73.8%, respectively) were shown. The area under the ROC curve (AUC) was 0.749 (95% CI: 0.693–0.806, *p* < 0.001)

The significant difference in SBA binding levels between patients with TAK and PAD prompted us to further explore the capability of SBA binding levels in the lectin microarray analysis as a potential biomarker to assist the differential diagnosis between TAK and PAD. Thus, ROC curve analysis was performed. As shown in Fig. 3, the AUC was 0.749 (95% CI: 0.693–0.806, *p* < 0.001), with a sensitivity of 73.8% and a specificity of 71.1% at the optimal cutoff value. These results suggested that the change of SBA affinity for serum IgG might be a promising serological indicator to assist the discrimination between TAK and PAD.

## Discussion

In the present study, we investigated the glycosylation profiles of serum IgG using a lectin microarray technology among patients with TAK, patients with PAD, and HCs. The results showed that the binding levels of SBA were significantly higher in patients with TAK compared to DCs and HCs. For patients with active TAK, the affinity of MNA-M for serum IgG was significantly decreased compared with that for patients with inactive TAK. These glycosylation changes were validated by lectin blot, confirming the reliability of the lectin microarray analysis. To the best of our knowledge, this is the first study to characterize the glycosylation patterns of serum IgG in patients with TAK.

IgG is the major immunoglobulin associated with humoral immunity. The pathogenic involvement of B cells and autoantibodies in TAK has been supported by several studies. A histological examination of the aortic wall specimens from patients with TAK has revealed massive B cell infiltration in the inflamed arterial adventitia [24]. When analyzing proportional changes of lymphocyte subsets in the peripheral blood, it has been observed that the frequency of B cell subsets in patients with TAK is higher than that in HCs [25]. Moreover, increased levels of AECAs in the culture supernatant of circulating B-lymphocytes have been observed in patients with TAK compared with that in HCs [6]. Most importantly, autoantibodies recognizing two distinct autoantigens that are specifically expressed on endothelial cells (ECs) in patients with TAK—endothelial protein C receptor and scavenger receptor class B type 1—have been identified and suggested as possible triggers of vascular inflammation [26]. Through binding to autoantigens on ECs, the autoantibodies are considered to disrupt the barrier function of the endothelial layer and facilitate the infiltration of immune cells, thereby promoting vascular inflammation [26].

Meanwhile, IgG is the most abundant glycoprotein in human serum and has N-linked oligosaccharides attached to the highly conserved asparagine 297 of the CH2 domain within each fragment crystallizable (Fc) region [27]. Variations in Fc glycans can affect the IgG affinity for Fc gamma receptors (FcγR) and components of complement systems [28], thereby having a large influence on the effector functions of IgG such as antibody-dependent cellular cytotoxicity (ADCC), complement-dependent cytotoxicity (CDC), and antibody-dependent cellular phagocytosis [29]. To date, aberrant IgG glycosylation has been recognized in a variety of autoimmune diseases and was demonstrated to impact the occurrence and development of diseases. For instance, decreased IgG Fc galactosylation and a concomitant increase of terminal *N*-acetylglucosamine (GlcNAc) residues have been strongly associated with RA [9], and these variations are found to make terminal GlcNAc residues more accessible for mannose-binding proteins, which results in enhanced activation of the complement system [30]. Studies have indicated that the removal of glycans at asparagine 297 of IgG CH2 domains reduces anti-inflammatory activity in SLE [31, 32]. Similarly, the removal of N-glycans of IgG from patients with immune thrombocytopenia has been implicated to reduce antibody-induced complement activation as well as platelet phagocytosis by monocytes, thereby increasing platelet survival in vivo [33].

In this study, significantly increased GalNAc glycosylation (recognized by SBA) of IgG in patients with TAK was observed compared with patients with atherosclerosis and HCs, which revealed a disease-specific glycan variation in TAK. This finding may have important clinical implications. Since there are some overlaps in the clinical and radiological features of TAK and atherosclerosis, the differential diagnosis can be quite difficult in some patients. Inflammatory indicators are not discriminative enough, as vessel wall inflammation and angiographic progression can persist despite normal inflammatory indicators [34], and atherosclerosis can sometimes present with elevated inflammatory indicators. In addition, although thought to be a sensitive and reliable tool to identify inflammation in large vessels [35], PET-CT is not an optimal tool to distinguish TAK from atherosclerosis, as focal or diffuse uptake can be observed in atherosclerotic lesions [36]. Therefore, more specific biomarkers are needed. Our results indicated that IgG GalNAc levels may be a useful diagnostic index for distinguishing individuals with TAK from atherosclerosis with satisfactory performance. Notably, no significant difference in GalNAc levels between patients with active and inactive TAK was identified, which indicated that the specific IgG glycoforms might be permanently encoded within B cells, resulting in sustained production of inflammatory glycans.

GalNAc is the most common linker of O-linked glycosylation [37]. Information regarding IgG O-linked glycan variations in autoimmune diseases is scarce. A pathogenic role of IgG O-glycosylation was supported by a study of RA where an association of decreased GalNAc levels and a clinical amelioration of the disease was observed [38]. It has been recognized that IgG O-glycans shield the hinge region from proteolytic digestion [39]. Therefore, aberrant GalNAc glycosylation is likely to exert its destructive effect by inhibiting the pathogenic IgG to be cleared and extending its half-life.

We further found that glycosylation features of serum IgG had significantly changed between patients with active and inactive TAK. Specifically, the mannose level (recognized by MNA-M) was decreased in patients with active TAK. The statistical correlation between the reduced mannose level and elevated inflammatory indicators also confirmed the decreasing mannose level to be a marker of disease activity. The difference in ConA binding levels between different disease states of TAK was not validated. One possible reason was that ConA could specifically bind not only mannose but also glucose. The binding levels of ConA were simultaneously affected by the glucose levels of serum IgG. Therefore, the binding levels of ConA did not entirely reflect mannose levels of serum IgG. It has been reported that IgG containing high mannose residues clears more rapidly from human serum because of more bindings to mannose receptors, which facilitate the uptake of IgG complexes by macrophages and dendritic cells [40]. Therefore, decreased high-mannose glycans of IgG in patients with active TAK might lead to a prolonged half-life of pathogenic IgG, thus causing persistent destruction to vascular ECs. Intriguingly, this finding is consistent with a previous study that reported higher AECAs levels in patients with active TAK compared to patients with inactive disease [6].

Furthermore, decreased mannose residues in IgG have been reported to exhibit reduced ADCC activity and decreased affinity for the FcγRIIIa, while the CDC activity was enhanced due to the increased C1q binding affinity [41, 42]. The possible mechanism is the relatively increased fucosylation in the glycan core structure. This is also in line with previous findings. It has been demonstrated that AECAs induce the apoptosis of ECs through CDC in TAK [43]. Moreover, Chauhan et al. found that the serum of patients with TAK could only induce apoptosis of aortic ECs in the presence of AECAs [44]. Our results indicated that serum IgG from patients with active TAK might have higher CDC activity and thus causing more serious damage to vascular ECs. Overall, we assumed that the serum IgG of patients with active TAK was pro-inflammatory and had a longer half-life. These results suggested that correcting the aberrant mannosylation of serum IgG in TAK might be able to accelerate the clearance of pathogenic IgG and inhibit the detrimental CDC activity, which might serve as a therapeutic target for TAK in the future.

There are several limitations to our study. First, although we used lectin blot to validate the specific glycan changes of serum IgG, these variations should be confirmed in a larger multi-center cohort of patients in the future. Second, the present study only quantified the glycosylation levels of serum IgG by lectin microarray without revealing the changes in the relative proportion of each oligosaccharide on IgG. Therefore, further studies are needed to elucidate the combined effects of altered glycans of serum IgG on the pathogenesis of TAK.

## Conclusions

The present study firstly characterized the changes in the glycosylation patterns of serum IgG in a large cohort of patients with TAK using a simple and high-throughput microarray-based strategy. Our results indicated that GalNAc levels were increased in patients with TAK compared to DCs and HCs, and the mannose levels were decreased in patients with active TAK relative to inactive TAK. We also showed that GalNAc levels of serum IgG have an important clinical value as a potential diagnostic indicator. Moreover, the mannose levels of serum IgG could reflect the disease activity of TAK. Therefore, our study provided new insights into the role of IgG in the pathogenesis of TAK and identified meaningful disease-related predictors for clinical applications.

## Supplementary Information

**Additional file 1: Figure S1. **The layout of the lectin microarray.

**Additional file 2: Figure S2.** Comparison of MNA-M binding levels of serum IgG in TAK subgroups

**Additional file 3: Table S1.** The glycan-binding specificities of the lectins contained on the lectin microarray.

## Data Availability

The datasets used and analyzed during the current study are available from the corresponding authors on reasonable request.

## Abbreviations

ADCC: Antibody-dependent cellular cytotoxicity
AECAs: Anti-endothelial cell antibodies
AUC: Area under the ROC curve
(b)DMARDs: (Biological) disease-modifying antirheumatic drugs
CAS: Carotid artery stenosis
CDC: Complement-dependent cytotoxicity
CLTI: Chronic limb-threatening ischemia
ConA: Concanavalin A lectin
DCs: Disease controls
ECs: Endothelial cells
ESR: Erythrocyte sedimentation rate
Fc: Fragment crystallizable
FcγR: Fc gamma receptors
FC: Fold change
GalNAc: *N*-Acetylgalactosamine
GlcNAc: *N*-Acetylglucosamine
HCs: Healthy controls
hsCRP: Hypersensitive C-reactive protein
IgG: Immunoglobulin G
IL-6: Interleukin-6
MNA-M: Morniga M lectin
ROC: Receiver-operating characteristic
PAD: Peripheral arterial disease
RA: Rheumatoid arthritis
S/N: Signal-to-noise ratio
SBA: Glycine max lectin
SDS-PAGE: Sodium dodecyl sulfate–polyacrylamide gel electrophoresis
SLE: Systemic lupus erythematosus
TAK: Takayasu arteritis

## References

[1] Rutter M, Bowley J, Lanyon PC (2021). A systematic review and meta-analysis of the incidence rate of Takayasu arteritis. Rheumatology (Oxford).

[2] Weyand CM, Goronzy JJ (2003). Medium- and large-vessel vasculitis. N Engl J Med.

[3] Berti A, Moura MC, Sechi E (2020). Beyond giant cell arteritis and Takayasu's arteritis: secondary large vessel vasculitis and vasculitis mimickers. Curr Rheumatol Rep.

[4] Watanabe R, Berry GJ, Liang DH (2020). Cellular signaling pathways in medium and large vessel vasculitis. Front Immunol.

[5] Gupta S (1981). Surgical and immunological aspects of Takayasu's disease. Ann R Coll Surg Engl.

[6] Wang H, Ma J, Wu Q (2011). Circulating B lymphocytes producing autoantibodies to endothelial cells play a role in the pathogenesis of Takayasu arteritis. J Vasc Surg.

[7] Schjoldager KT, Narimatsu Y, Joshi HJ (2020). Global view of human protein glycosylation pathways and functions. Nat Rev Mol Cell Biol.

[8] Goulabchand R, Vincent T, Batteux F (2014). Impact of autoantibody glycosylation in autoimmune diseases. Autoimmun Rev.

[9] van de Geijn FE, Wuhrer M, Selman MH (2009). Immunoglobulin G galactosylation and sialylation are associated with pregnancy-induced improvement of rheumatoid arthritis and the postpartum flare: results from a large prospective cohort study. Arthritis Res Ther.

[10] Vučković F, Krištić J, Gudelj I (2015). Association of systemic lupus erythematosus with decreased immunosuppressive potential of the IgG glycome. Arthritis Rheumatol.

[11] Youinou P, Pennec YL, Casburn-Budd R (1992). Galactose terminating oligosaccharides of IgG in patients with primary Sjögren's syndrome. J Autoimmun.

[12] Cvetko A, Kifer D, Gornik O (2020). Glycosylation alterations in multiple sclerosis show increased proinflammatory potential. Biomedicines..

[13] Miyoshi E, Shinzaki S, Fujii H (2016). Role of aberrant IgG glycosylation in the pathogenesis of inflammatory bowel disease. Proteomics Clin Appl.

[14] Selman MH, Niks EH, Titulaer MJ (2011). IgG fc N-glycosylation changes in Lambert-Eaton myasthenic syndrome and myasthenia gravis. J Proteome Res.

[15] Hirabayashi J, Yamada M, Kuno A (2013). Lectin microarrays: concept, principle and applications. Chem Soc Rev.

[16] Arend WP, Michel BA, Bloch DA (1990). The American College of Rheumatology 1990 criteria for the classification of Takayasu arteritis. Arthritis Rheum.

[17] Kerr GS, Hallahan CW, Giordano J (1994). Takayasu arteritis. Ann Intern Med.

[18] Cimminiello C (2002). PAD Epidemiology and pathophysiology. Thromb Res..

[19] Aboyans V, Ricco JB, Bartelink MEL (2018). 2017 ESC Guidelines on the Diagnosis and Treatment of Peripheral Arterial Diseases, in collaboration with the European Society for Vascular Surgery (ESVS): Document covering atherosclerotic disease of extracranial carotid and vertebral, mesenteric, renal, upper and lower extremity arteriesEndorsed by: the European Stroke Organization (ESO)The Task Force for the Diagnosis and Treatment of Peripheral Arterial Diseases of the European Society of Cardiology (ESC) and of the European Society for Vascular Surgery (ESVS). Eur Heart J.

[20] Silver JD, Ritchie ME, Smyth GK (2009). Microarray background correction: maximum likelihood estimation for the normal-exponential convolution. Biostatistics.

[21] Hu C, Zhang P, Li L (2021). Assessing serum IgG4 glycosylation profiles of IgG4-related disease using lectin microarray. Clin Exp Rheumatol.

[22] Jennewein MF, Alter G (2017). The Immunoregulatory Roles of Antibody Glycosylation. Trends Immunol.

[23] Prozeller D, Rosenauer C, Morsbach S (2020). Immunoglobulins on the surface of differently charged polymer nanoparticles. Biointerphases.

[24] Inder SJ, Bobryshev YV, Cherian SM (2000). Immunophenotypic analysis of the aortic wall in Takayasu's arteritis: involvement of lymphocytes, dendritic cells and granulocytes in immuno-inflammatory reactions. Cardiovasc Surg.

[25] Hoyer BF, Mumtaz IM, Loddenkemper K (2012). Takayasu arteritis is characterised by disturbances of B cell homeostasis and responds to B cell depletion therapy with rituximab. Ann Rheum Dis.

[26] Mutoh T, Shirai T, Ishii T (2020). Identification of two major autoantigens negatively regulating endothelial activation in Takayasu arteritis. Nat Commun.

[27] Arnold JN, Wormald MR, Sim RB (2007). The impact of glycosylation on the biological function and structure of human immunoglobulins. Annu Rev Immunol.

[28] Quast I, Peschke B, Lünemann JD (2017). Regulation of antibody effector functions through IgG Fc N-glycosylation. Cell Mol Life Sci.

[29] Jefferis R (2009). Glycosylation as a strategy to improve antibody-based therapeutics. Nat Rev Drug Discov.

[30] Malhotra R, Wormald MR, Rudd PM (1995). Glycosylation changes of IgG associated with rheumatoid arthritis can activate complement via the mannose-binding protein. Nat Med.

[31] Lood C, Allhorn M, Lood R (2012). IgG glycan hydrolysis by endoglycosidase S diminishes the proinflammatory properties of immune complexes from patients with systemic lupus erythematosus: a possible new treatment?. Arthritis Rheum.

[32] Albert H, Collin M, Dudziak D (2008). In vivo enzymatic modulation of IgG glycosylation inhibits autoimmune disease in an IgG subclass-dependent manner. Proc Natl Acad Sci U S A.

[33] Bakchoul T, Walek K, Krautwurst A (2013). Glycosylation of autoantibodies: insights into the mechanisms of immune thrombocytopenia. Thromb Haemost.

[34] Mason JC (2010). Takayasu arteritis–advances in diagnosis and management. Nat Rev Rheumatol.

[35] Balink H, Bennink RJ, van Eck-Smit BL (2014). The role of 18F-FDG PET/CT in large-vessel vasculitis: appropriateness of current classification criteria?. Biomed Res Int.

[36] James OG, Christensen JD, Wong TZ (2011). Utility of FDG PET/CT in inflammatory cardiovascular disease. Radiographics.

[37] Kaur H (2021). Characterization of glycosylation in monoclonal antibodies and its importance in therapeutic antibody development. Crit Rev Biotechnol.

[38] Stümer J, Biermann MHC, Knopf J (2017). Altered glycan accessibility on native immunoglobulin G complexes in early rheumatoid arthritis and its changes during therapy. Clin Exp Immunol.

[39] Plomp R, Dekkers G, Rombouts Y (2015). Hinge-region O-glycosylation of human immunoglobulin G3 (IgG3). Mol Cell Proteomics.

[40] Goetze AM, Liu YD, Zhang Z (2011). High-mannose glycans on the Fc region of therapeutic IgG antibodies increase serum clearance in humans. Glycobiology.

[41] Kanda Y, Yamada T, Mori K (2007). Comparison of biological activity among nonfucosylated therapeutic IgG1 antibodies with three different N-linked Fc oligosaccharides: the high-mannose, hybrid, and complex types. Glycobiology.

[42] Zhou Q, Shankara S, Roy A (2008). Development of a simple and rapid method for producing non-fucosylated oligomannose containing antibodies with increased effector function. Biotechnol Bioeng.

[43] Tripathy NK, Upadhyaya S, Sinha N (2001). Complement and cell mediated cytotoxicity by antiendothelial cell antibodies in Takayasu's arteritis. J Rheumatol.

[44] Chauhan SK, Tripathy NK, Nityanand S (2006). Antigenic targets and pathogenicity of anti-aortic endothelial cell antibodies in Takayasu arteritis. Arthritis Rheum.

