# Supplementary figures and images for: Altered glycosylation profiles of serum IgG in Takayasu arteritis
Source: Eur J Med Res. 2023 Feb 8;28:69. doi: 10.1186/s40001-023-01035-4 (PMC9906894; doi:10.1186/s40001-023-01035-4)

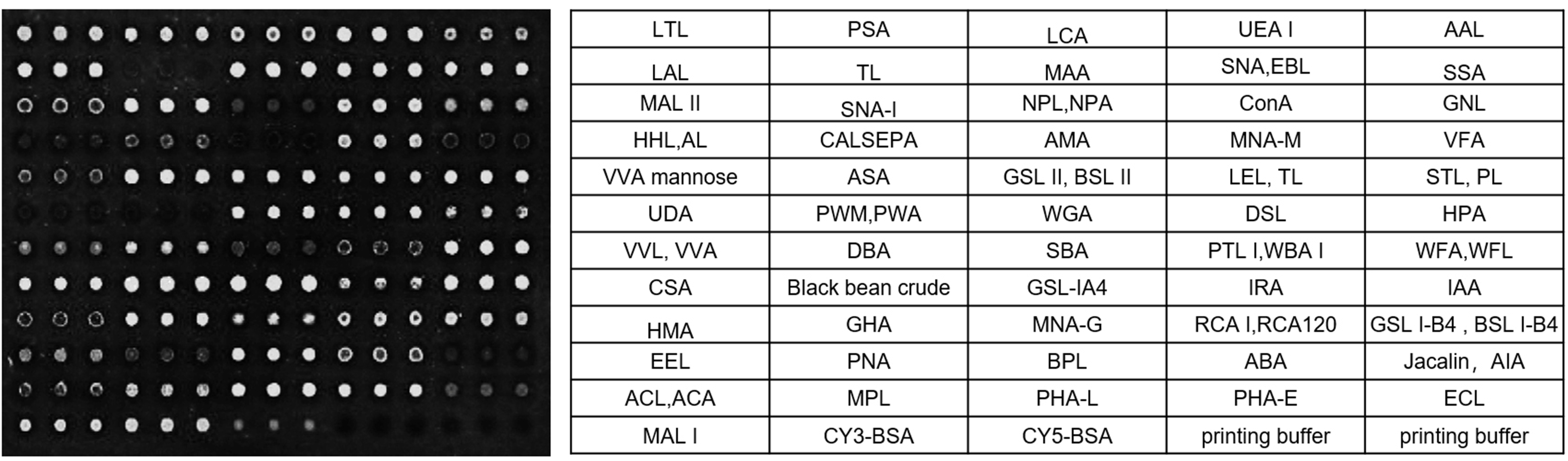

Supplement: Supplementary file 1 — Additional file 1: Figure S1. The layout of the lectin microarray. [file 40001_2023_1035_MOESM1_ESM.jpg]

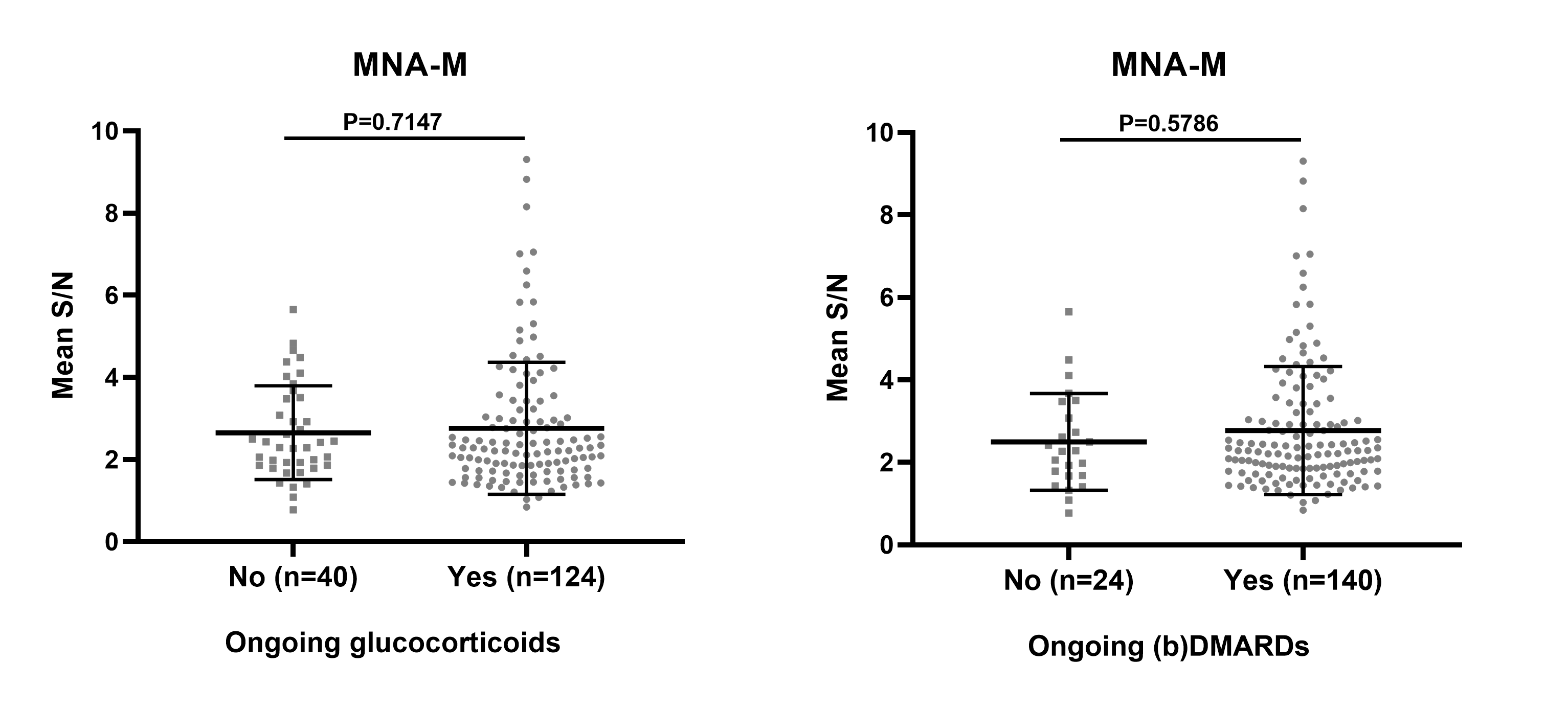

Supplement: Supplementary file 2 — Additional file 2: Figure S2. Comparison of MNA-M binding levels of serum IgG in TAK subgroups [file 40001_2023_1035_MOESM2_ESM.tif]
